# Review of Ginseng Anti-Diabetic Studies

**DOI:** 10.3390/molecules24244501

**Published:** 2019-12-09

**Authors:** Wei Chen, Prabhu Balan, David G. Popovich

**Affiliations:** 1 School of Food and Advanced Technology, Massey University, Palmerston North 4442, New Zealand; W.Chen2@massey.ac.nz; 2 Riddet Institute, Massey University, Palmerston North 4442, New Zealand; P.Balan@massey.ac.nz; 3 Alpha-Massey Natural Nutraceutical Research Centre, Massey University, Palmerston North 4442, New Zealand

**Keywords:** ginseng, ginsenosides, anti-diabetes, insulin, blood glucose

## Abstract

Ginseng is one of the most valuable and commonly used Chinese medicines not only in ancient China but also worldwide. Ginsenosides, also known as saponins or triterpenoids, are thought to be responsible for the beneficial effects of ginseng. In this review, we summarize recent publications on anti-diabetic studies of ginseng extracts and ginsenosides in cells, animals, and humans. It seems that the anti-diabetic effect of ginseng is positive for type 2 diabetic patients but has no significant impact on prediabetes or healthy adults. Regulation of insulin secretion, glucose uptake, anti-oxidative stress, and anti-inflammatory pathways may be the mechanisms involved with ginseng’s anti-diabetic effects. Taken together, this summary provides evidence for the anti-diabetes effects of ginseng extracts and ginsenosides as well as the underlying mechanisms of their impact on diabetes.

## 1. Introduction

The main type of diabetes is type 1 diabetes, which is caused by insulin deficiency, and type 2 diabetes, which is characterized by insulin resistance [1]. The majority of diabetes is type 2 diabetes, and it is a formidable challenge for public health [2]. According to the International Diabetes Federation statistics, 463 million people aged 20–79 years worldwide had type 2 diabetes in 2019, and this number is estimated to reach 700 million by 2045 [3]. There is also a huge population with prediabetes, which is very likely to develop into type 2 diabetes [4]. Without proper management, diabetes can cause serious health problems, especially diabetic complications. For example, it can damage eyes, kidneys, and nerves, and it also causes heart disease, stroke, and can even necessitate limb removal [5]. Thus, proper treatment of diabetes is important. Although many researchers are looking at ways to reduce insulin resistance to treat type 2 diabetes, type 2 diabetes is still a severe disease and cannot be cured [6]. Currently, management of diabetes mainly focuses on insulin or its peptide derivatives, anti-diabetic oral drugs, and diet control [7]. Insulin and its peptide derivative treatments need to be injected via vein every day, leading to inconvenience and burdens for patients [8]. Long-term oral administration of chemical drugs can be harmful for many diabetic patients because of drug toxicities [8]. Researchers are exploring the possibilities offered by complementary and alternative medicine, such as traditional herbal medicine.

There has been increasing popularity in Chinese medicine, especially since the 2015 Nobel Prize was awarded to Tu Youyou, who discovered that artemisinin from traditional Chinese medicine can treat malaria [9]. This popularity indicates that even in modern times, ancient Chinese herbal remedies can be valuable as an alternative therapy for relieving serious diseases. One of the Chinese medicinal herbs with promising anti-diabetic effects is ginseng [10]. In fact, ginseng has long been used to treat diabetes in China. In the Song Dynasty (1078 A.D.), official documentation (Formularies of the Bureau of People’s Welfare Pharmacies) recorded that ginseng was employed to cure Xiaoke disease, which is nowadays known as diabetes [10]. As a medicinal intervention management to diabetes, the Asian (*Panax ginseng*) and American (*Panax quinquefolius* L.) ginsengs are the two most widely used varieties and have recently attracted a lot of attention from those trying to understand the molecular mechanisms of ginseng’s anti-diabetic effects. 

**Figure 1:**
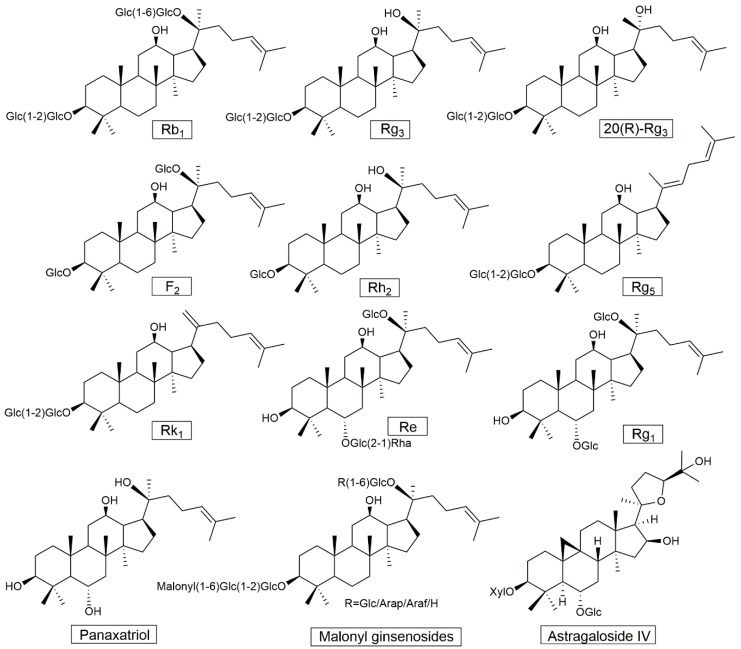
Chemical structures of triterpenoid saponins mentioned in this study. Glc, Rha, Arap, Araf, and Xyl refer to β-d-glucopyranosyl, α-l-rhamnopranosyl, α-l-arabinopyranosyl, α-l-arabinofuranosyl, and β-d-xylopyranosyl, respectively.

Ginseng contains diversified components, including saponins, polysaccharides, polyacetylenes, phenols, and alkaloids. Ginseng saponins, known as ginsenosides, are an important class of natural triterpene saponins, which are thought to be responsible for the anti-diabetic effect in ginseng. So far, nearly two hundred ginsenosides have been detected from ginseng plants and heat-processed ginseng products [11]. Most known ginsenosides are classified as members of the dammarane family, and consist of a four-ring, steroid-like structure, such as ginsenosides Rb1, Rg3, Re, and Rg1 (structures shown in Figure 1). Increasing studies suggest that ginseng extracts, processed ginseng extracts, and ginsenosides show anti-diabetic effects, although the mechanism of action is still not clear. A systematic summary of anti-diabetic studies of ginseng will be helpful to explore its anti-diabetic mechanism. 

Until 2011, there were eleven publications (twelve human trials) about ginseng extracts being used for diabetes intervention. Four of them studied American ginseng root extracts, five of them studied Asian ginseng root extracts, two used ginsenosides (Rb1 and Rg1, Re), and one tested eight types of ginseng (American, American-wild, Asian, Asian-red, Vietnamese-wild, Siberian, Japanese-rhizome, and Sanchi ginseng) on acute postprandial glycemic indices in healthy humans. Through summarizing these twelve human trials, along with in vitro cell studies and in vivo animal studies, the authors deduced that ginseng may modulate insulin production/secretion, glucose metabolism and uptake, or inflammatory pathway to exert anti-diabetic effects [12]. Recently, many studies, including human studies, in vitro and in vivo studies have been conducted on diabetes intervention with ginseng extracts or ginsenoside. In order to better know the progress and basis for the evaluation of the anti-diabetic effects of ginseng, this current review summarizes the research into type 2 diabetes intervention with ginseng extracts or ginsenosides conducted in human trials, in vivo animal studies, and in vitro cell studies since 2012. 

## 2. Anti-Diabetic Effects of Ginseng in Human Trials 

Although ginseng has been used as a natural herb medicine for a long time in East Asia, interest in the anti-diabetic efficacy of ginseng has drawn increased attention from modern medicine. An increasing number of human trials are exploring the efficacy of ginseng intervention in diabetes. Shishtar et al. found ginseng significantly reduced fasting blood glucose compared to the control (−0.31 mmol/L [95% CI: −0.59 to −0.03], *p* = 0.03) based on a meta-analysis of sixteen randomized controlled clinical trials; there was no significant effect on fasting plasma insulin and glycated hemoglobin [13]. Another meta-analysis included eight trials that suggested ginseng supplementation showed significant differences on fasting glucose (−0.306 mmol/L [95% CI: −0.539 to −0.074], *p* = 0.01), postprandial insulin (−2.132 mmol/L [95% CI: −3.706 to −0.558], *p* = 0.008), and HOMA-IR (−0.397 mmol/L [95% CI: −0.679 to −0.115], *p* = 0.006) compared to the control group; there was no significant difference on postprandial glucose and fasting insulin between ginseng treatment and control groups [14]. Since 2012, there have been 13 human trials published that have focused on the anti-diabetic effect of ginseng (Asian ginseng (*Panax ginseng*) and American ginseng (*Panax quinquefolius* L.)). Different ginseng extracts or differently processed ginseng products exhibited varied impact on diabetic patients. The information of ginseng extracts mentioned in this study is shown in Table 1. 

Ten clinical trials on Asian ginseng have been reported in recent years. In one trial, thirty-six diabetic patients were randomized to receive 1.5 g/day of ginsam, which is enriched ginsenoside Rg3, though a vinegar extraction from *P. ginseng*, or a daily placebo for eight weeks. HbA1c levels and fasting blood glucose in the ginsam-treated group significantly reduced by 0.56% and 21.40 mg/dL compared to the placebo group [15]. Park et al. reported that 23 impaired fasting glucose participants were randomly administrated with 960 mg/day of hydrolyzed Asian ginseng extract or a placebo for eight weeks. The hydrolyzed ginseng extract, containing 7.54 mg/g of Rg1, 6.30 mg/g of compound K, 5.42 mg/g of Rb1, 1.87 mg/g of Re, 0.70 mg/g of Rd, 0.36 mg/g of Rb2, and 0.29 mg/g of Rc, significantly decreased fasting plasma glucose (*p* = 0.017) and postprandial glucose (*p* = 0.01) compared to the placebo but there were no significant differences in fasting plasma insulin and postprandial insulin between the groups [16]. These results showed that hydrolyzed ginseng extract cut the absorption of glucose in the intestinal lumen. Further large-scale and long-term studies are needed to fully evaluate whether it can decrease insulin secretion. 

The ginseng berry has a different ginsenoside composition and contains more plentiful ginsenosides than its root [17]. An in vivo study using *ob/ob* diabetic mice reported that after being treated with 150 mg/kg ginseng berry extract, fasting blood glucose significantly decreased to 150 mg/dL on day 5 and 129 mg/dL on day 12. The same experiment was carried out with ginseng root extract. Although the same dose of ginseng root extract significantly reduced the fasting blood glucose to 143 mg/dL on day 5, the fasting blood glucose had not decreased by day 12 (155 mg/dL) [18]. This study showed that ginseng berry extract exhibited more potent anti-hyperglycemic effects compared to ginseng root extract administrated at the same concentrations using *ob/ob* diabetic mice. Recently, a 12-week, randomized, double-blind, placebo-controlled clinical trial was completed with 72 participants with a fasting glucose level ranging from 100–140 mg/dL. The study showed that ginseng berry extract significantly reduced fasting blood glucose and postprandial glucose at 60 min in an oral glucose tolerance test after a 12-week treatment, but it did not regulate serum glucose to normal levels [19], indicating that the ginseng berry extract failed to show anti-hyperglycemic effects in this human study. It should be noted that the participants in this study were prediabetes rather than type 2 diabetic patients. The anti-hyperglycemic effect was observed in the *ob/ob* diabetic mice model [18]. Maybe the anti-hyperglycemic effect of ginseng berry extract is positive for type 2 diabetes rather than prediabetes. Further investigation of people with type 2 diabetes is needed to test this hypothesis. 

Participants with impaired fasting glucose, impaired glucose tolerance, or type 2 diabetes consuming 5 g/day Korean red ginseng (KRG) extract were found to have significantly reduced blood glucose. Specifically, −1.23 ± 0.59 mmol/L of serum glucose and –0.97 ± 0.30 mmol/L of whole blood glucose were decreased at a 30 min oral glucose tolerance test after a 12-week intervention [20]. The plasma insulin sensitivity index increased by 33% compared to the placebo group in this 12-week randomized, double-blind and placebo-controlled trial [20]. 

In a randomized, double-blind, placebo-controlled trial involving forty-two subjects, 2.7 g/day of fermented red ginseng given for four weeks significantly increased postprandial insulin of subjects with impaired fasting glucose or type 2 diabetes (35.5 µU/mL in the placebo group vs. 56.3 µU/mL in the ginseng group, *p* = 0.040). Moreover, in the ginseng treatment group, their postprandial glucose and glucose area under curves (AUC) significantly reduced by 17.2% (*p* = 0.0001) and 27.4% (*p* = 0.002) compared to the baseline values. However, the ginseng treated group did not show a remarkable difference in fasting blood glucose and insulin relative to the placebo [21]. It is known that fermentation can increase the bioavailability (absorption and bioactivity) of ginseng due to small ginsenosides (ginsenoside Rg3, compound K, etc.) produced through the fermentation process. As reported by Bang et al. in the clinical trial [20], 5 g/day of Korean red ginseng can reduce fast blood glucose and glucose AUC. However, in this study [21], the fermented red ginseng only modulated postprandial glucose and insulin, not fasting glucose and insulin. The short period of time (four weeks) may be responsible for the lack of significant differences in fasting glucose and insulin because the improvement of fasting glucose and insulin is usually expected over a long-term treatment, while postprandial glucose and insulin are more sensitive to slight interventions in glycemic control. Long-term clinical trials for fermented red ginseng are needed. 

These studies suggest that different Asian ginseng extracts consistently reduce the fasting blood glucose or postprandial glucose in type 2 diabetic patients and could be considered as an optional therapy for managing type 2 diabetes. However, other results from human studies cast doubts about this view. 

Sixty-eight obese subjects without diabetes were administrated 6 g/day of KRG (orally) or a placebo for 12 weeks in a randomized, double-blind, and placebo-controlled trial. This study showed that there was no significant effect on the insulin level and the insulin sensitivity index between the ginseng treatment group and placebo group [22]. Another randomized, double-blind, placebo-controlled 8-week trial conducted among fifty obese women found that there were significant improvements in the obesity index between before and after treatment with 6 g/day KRG, but no significant difference between the KRG treatment group and the placebo group [23]. Both of these studies indicate that KRG does not enhance insulin sensitivity in obese people without diabetes. In both trials, the regulation of glucose homeostasis was not observed in the prediabetes situation for subjects who were obese without diabetes, impaired glucose tolerance or mild type 2 diabetes. Recently, a multicenter, double-blind, randomized, and placebo-controlled trial was conducted on 1000 healthy adults. The test group consumed 2 g/day of KRG for 24 weeks, which was found to be safe and well-tolerated in healthy adults, and there were no significant abnormal changes from anthropometric, laboratory, and vital sign measurements between the KRG group and the placebo group [24]. Therefore, it seems that the anti-diabetic effect of Asian ginseng is positive for type 2 diabetic patients, but there has no significant effect on prediabetes or healthy adults. 

Besides the human trials using Asian ginseng, there have been three trials using American ginseng on type 2 diabetic patients. In one study, thirty type-2 patients were involved in a randomized, placebo-controlled crossover trial. HbA1c levels were 0.31% lower (*p* = 0.011) after treatment with 6 g konjac-glucomannan-based fiber blended together with 3 g American ginseng per day for 12 weeks. Plasma lipids of the ginseng intervention group also significantly decreased compared to the placebo control group [25]. This study did not consider the effect of konjac-glucomannan-based fiber on diabetes. The study did not report whether the konjac-glucomannan-based fiber blend or the American ginseng was responsible for reducing the glucose levels in the trial subjects. The study could be improved by planning another group administrated with konjac-glucomannan-based fiber, or adding it to the placebo. Vuksan et al. [26] conducted a randomized, double-blind, and placebo-controlled crossover clinical study to assess the anti-diabetic efficacy and safety of American ginseng. Twenty-four individuals with well-controlled type 2 diabetes completed the study. Using a double-blind, crossover design, the participants were randomized to receive either 3 g/day of American ginseng extract or a placebo for eight weeks together with their original treatment. The efficacy was assessed by HbA1c, and safety was assessed by liver and kidney function testing. The researchers found that American ginseng significantly reduced HbA1c levels (−0.29%, *p* = 0.041), fasting blood glucose (−0.71 mmol/L, *p* = 0.008), and systolic blood pressure (−5.6 mmHg, *p* < 0.001) compared to the placebo. Furthermore, the beneficial changes did not affect the safety profiles. Another human study specifically tested the safety of American ginseng as an adjunct to conventional therapy in type 2 diabetes [27]. Seventy-four type 2 diabetic patients were given 3 g/day American ginseng extract or a placebo for 12 weeks. The investigators found that there was no significant difference between the ginseng treatment and the placebo in the safety parameters, such as kidney function (urates and creatinine), liver function (AST and ALT), and hemostatic function (PT and INR). These studies suggest that American ginseng extract is effective and safe as an additional treatment in the management of type 2 diabetes. 

Together, these human studies show that both Asian ginseng and American ginseng do indeed decrease blood glucose in type 2 diabetics. At the same time, it should be noted that this efficacy was observed in several limited ginsenosides (6–7 ginsenosides analyzed), small sample sizes (23–94 subjects), and short-term studies (4–12 weeks). Larger scale clinical trials are needed to completely illuminate the long-term benefits of this herbal supplement in the management of type 2 diabetes. The studies are summarized in Table 2. 

## 3. Potential Mechanisms of the Anti-Diabetic Effect of Ginseng

Apart from the clinical evidence for the anti-diabetic effect of ginseng, there have also been animal studies conducted to explore the underlying mechanisms of the anti-diabetic effect of ginseng, along with some cell experiments in vitro. Different processed ginseng extracts and some individual ginsenosides (structures shown in Figure 1) have been reported to exhibit an anti-diabetic effect in cell and animal experiments (Table 3 and Table 4). Although the anti-diabetic mechanism of ginseng has not been entirely clarified, the available data indicate that the regulation of blood glucose by ginseng is possibly related to the following four aspects.

First, ginseng modulates blood glucose levels by improving β-cell function and enhancing insulin sensitivity. *P. ginseng* berry extract increased β-cell proliferation and insulin secretion to improve glycemic control in streptozotocin (STZ) -induced diabetic mice [28]. Ginseng berry extract could also improve insulin sensitivity in C57BL/6 mice over 15 months old, likely by increasing the activation of IRS-1 and AKT [29]. Kim et al. found that 200 mg/kg of black ginseng extract inhibited β-cell apoptosis and improved islet architecture, which led to the enhancement of β-cell function and reduction of hyperglycemia in STZ-treated mice [30]. While exploring the anti-diabetic component of ginseng extracts, both PPD-type ginsenosides and PPT-type ginsenosides were investigated in animal studies. Ginsenoside Rb1, one of the main PPD-type ginsenosides, was found to reduce symptoms of decreased insulin sensitivity and elevated blood glucose caused by the high-fat diet induction of type 2 diabetic mice [31]. Using the STZ-induced diabetic rat model, researchers found that a main PPT-type ginsenoside, Rg1, can lower insulin resistance and blood glucose, and also improve the blood lipid profile and liver function [32], suggesting that Rg1 may be a potential adjuvant therapy for type 2 diabetic patients with fatty liver disease. As well as the main components in fresh ginseng extracts, heat-treated ginsenoside Rg3 has also been tested. Ginsenoside Rg3 is the main metabolite degraded from other abundant ginsenosides during heat-processing fresh ginseng to manufacture red ginseng and black ginseng. Kim et al. screened the GLP-1 release ability of 15 ginsenosides and found that Rg3 showed the strongest GLP-1 secretion (about 27 pM/mg) effect in NCI-H716 cells. In this in vivo trial, 10 µM Rg3 significantly raised production of GLP-1 and insulin to reduce blood glucose in *db/db* mice [33]. In mouse islet cells, insulin secretion was significant: 2.3 times higher in the 4 μM Rg3 treatment group compared to control group [34]. Taken together, Rg3 is presumably the main active anti-diabetic ingredient of ginseng, although administration with other ginsenosides has also shown hypoglycemic effects; these ginsenosides can be hydrolysed to Rg3 in the gastrointestinal tract [35] to further exert pharmacological effects.

Second, ginseng can enhance glucose uptake by up-regulating the expression of glucose transporters (GLUT). One percent Fermented red ginseng (FRG) significantly decreased the body weight of *ob/ob* mice compared with the controls after 4 weeks of administration during the 16-week treatment period; blood glucose was also significantly reduced after 16-weeks treatment with FRG [44]. Furthermore, the study found that the expressions of GLUT1 and GLUT4 were significantly up-regulated by FRG in liver and muscle. This suggests that FRG may decrease blood glucose through enhancing glucose uptake in skeletal muscle. Similar results were also observed in *db/db* mice treated with ginseng extracts. 0.5 g/kg fermented steam-dried ginseng berry (FSGB) [45] significantly decreased body weight and blood glucose in *db/db* mice during a 35-day treatment; furthermore, the plasma insulin in the FSGB treated group (17.05 ± 0.49 ng/mL) significantly increased almost double compared to the untreated *db/db* group (8.21 ± 0.60 ng/mL). GLUT1 mRNA expression was up-regulated by FSGB in the L6 skeletal muscle cells. During an 8-week treatment with 0.1% fermented ginseng extract (FGE) in *db/db* mice, the body weight, fasting blood glucose, and HbA1c level significantly (*p* < 0.05) decreased compared to control groups [46]. FGE intervention significantly enhanced the mRNA expression of GLUT2 and lowered the glucose-6-phosphotase (G6Pase) expression in liver tissues [46]. G6Pase plays an important role in glucose homeostasis because it is a main enzyme of gluconeogenesis and glycogenolysis in the liver. The reduction of G6Pase and increase of GLUT2 expressions mean FGE may improve the transport of blood glucose into the liver, which leads to decreasing the blood glucose level in *db/db* mice. Moreover, the FGE group exhibited a higher level of insulin secretion than the control group in the islet primary cell culture; the FGE treated group had weaker damage and inflammation than the control group in vivo hematoxylin–eosin staining assay [46]. These suggest that FGE has a protective effect on the pancreas and improves the production/secretion of insulin. Kang et al. found that 900 mg/kg black ginseng ethanol extract (GBG05-FF) significantly reduced fasting blood glucose, glucose tolerance, and plasma HbA1c after four weeks of treatment. Furthermore, GBG05-FF up-regulated the expressions of GLUT2 in the liver and GLUT4 in the muscles [43], indicating that black ginseng can enhance glucose uptake in surrounding tissues to regulate blood glucose levels. In addition, other beneficial physiological changes, such as a reduction of total cholesterol and triglycerides, and increased PPAR-γ and PEPCK were also observed after administration with fermented ginseng berry [46] or black ginseng [43]. Ginsenosides Rg1 [66] and Re [67] increased expressions of GLUT4 through AMPK pathways in C2CI2 muscle cells and via increasing PPAR-γ activity in 3T3-L1 cells, respectively. Furthermore, KRG at a dose of 0.2 g/kg/d for a 12-week treatment period enhanced insulin action and secretion in diabetic Goto-Kakizaki rats by up-regulating the GLUT4 expression in adipose tissue and down-regulating the expressions of UCP2 and PARP in the pancreas, and PTP-1B in adipose tissue and skeletal muscle [40].

Third, suppression of oxidative stress through increasing SOD activity and decreasing MDA production was reported to occur in treatment with either ginseng polysaccharides in STZ-induced ICR diabetic mice [50], or ginsenoside Rg1 in STZ-induced diabetic rats [57]. Administration of GS-E3D (pectin lyase-modified red ginseng extract) for six weeks significantly decreased urinary levels of albumin, 8-hydroxy-2′-deoxyguanosine (8-OHdG), and advanced glycation end-products (AGEs) in STZ-induced diabetic rats. Moreover, all symptoms of diabetic nephropathy were improved by GS-E3D treatment via suppressing renal accumulation of AGEs and oxidative stress [47]. Tissue culture raised mountain ginseng adventitious root (TCMGARs) extracts at dosage levels of 250, and 500 mg/kg significantly reduced the blood glucose, total cholesterol, and triglyceride content in STZ-induced diabetic rats [38]. In an LDL^-/-^ mouse model, Rg3-enriched red ginseng extract (44.91 mg/g, 66.6% occupy for total ginsenosides) significantly decreased the levels of glucose, triglyceride, low-density lipoprotein, alanine aminotransferase and aspartate aminotransferase compared to the control [48]. In a high-fat induced C57BL/6 mouse model, ginsenoside Re significantly reduced fasting blood glucose levels and related biochemical parameters, including cholesterol, low-density lipoprotein cholesterol, total triglyceride, glutamic-pyruvic transaminase, and glutamic-oxaloacetic transaminase. Re also regulated the level of ACh, AChE, and oxidative stress-related parameters (MDA, SOD, GSH) via the JNK pathway [55]. 

In addition to the above three potential anti-diabetic mechanisms, ginseng can also show anti-diabetic activity through other pathways. Several animal studies have found that ginseng processed products or individual ginsenosides regulate blood glucose in diabetic mouse or rat models, accompanied by regulating the expression of TNF-α, eNOS [46,53,56], suggesting that diabetes is associated with inflammation, and effective modulation by ginseng on inflammation may be able to prevent the development of insulin resistance. Recently, Shen et al. reported that Rg1 released in brain tissue using nano-drug delivery systems reduced the volume of cerebral infarction and improved neural recovery in diabetic rats with cerebral infarction [59], indicating that the hypoglycemic effect of Rg1 may be regulated through the central nervous system. Additionally, in an STZ-induced type 1 diabetic rat model, ginsenoside Rh2 reduced fasting blood glucose and improved cardiac function via enhancing PPAR-δ signaling in diabetic rats with cardiac fibrosis [60]. In the 3T3-L1 cells, researchers that found ginsenoside F2 [68], and a ginsenosides Rg5 + Rk1 mixture [69] could reduce lipid accumulation and down-regulate expression of PPAR-γ.

**Figure 2:**
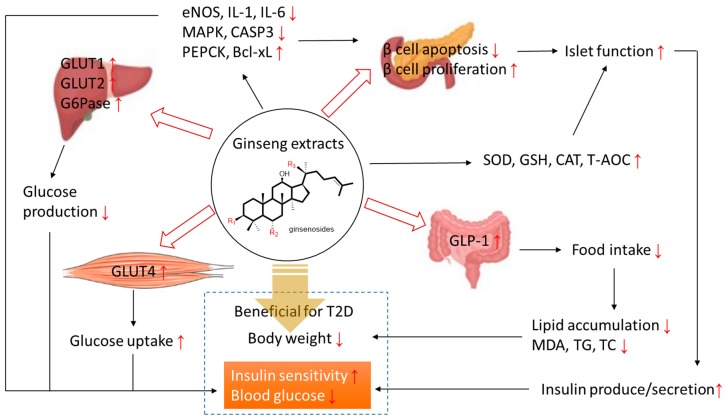
The potential mechanism of ginseng extracts (ginsenosides) on type 2 diabetes. Bcl-xL: B-cell lymphoma-extra-large; CASP3: caspase-3; CAT: catalase; eNOS: endothelial nitric oxide synthase; G6Pase: glucose-6-phosphotase; GLP-1: glucagon-like peptide-1; GLUT: glucose transporter protein; GSH (-PX): glutathione peroxidase; IL: interleukin; MAPK: mitogen-activated protein kinase; MDA: malondialdehyde; PEPCK: phosphoenolpyruvate carboxykinase; SOD: superoxide dismutase; T-AOC: total anti-oxidative capacity; TC: total cholesterol; TG: triglycerides.

Based on studies on diabetes intervention with ginseng in vivo and in vitro, the potential mechanism of ginseng extracts (ginsenosides) on type 2 diabetes is summarized in Figure 2. 

## 4. Conclusions

Human, animal, and cell studies have shown that different processed ginseng extracts and specific ginsenosides possess beneficial effects on diabetes, especially type 2 diabetes. Most studies of individual ginsenosides have focused on Rb1, Re, or Rg1, which are the main components of ginseng and are easily obtained. However, these ginsenosides have a large molecule structure, resulting in poor systemic bioavailability. Reeds et al. reported on overweight and obese participants with impaired glucose tolerance or newly diagnosed type 2 diabetes who consumed ginseng root extract (8 g/day), ginsenoside Re (250–500 mg/day), or placebo for 30 days; ginsenosides Rb1, Rb2, and Re were not detected in plasma after treatment with ginseng root extract or ginsenoside Re [70]. However, another study conducted in healthy volunteers showed that ginsenoside Re and its potential metabolites (including Rg2, F1, Rh1, and PPT) were detected in plasma after oral administration of ginsenoside Re [71]. It seems that the large molecule ginsenosides (Rb1, Rc, Re) may be a form of storage for saponins in ginseng plants rather than the active form in vivo. The related but smaller molecule ginsenosides (Rg3, Rh1) may be the ingredient that exerts therapeutic effects. This is also supported by the evidence found in experiments with red ginseng, fermented ginseng, and black ginseng. Therefore, the metabolic ginsenosides (Rg3, Rh1) need to be investigated to determine the active form of ginsenosides in vivo. 

Another concern is that the results from clinical data for different processed ginseng extracts are inconsistent; some studies showed that the ginseng extracts possessed anti-hyperglycemic or diabetes-related effects, while others did not. These results could be caused by subject factors and/or drug reasons. The physical activity, body weight, diabetic degree, and sample size of volunteers may affect the outcomes of clinical trials. Another influencing factor is the variability of ginseng extracts. Different sources, species, or extraction processes lead to different ginseng ingredients. Even when using the same species and the same extraction process, different batches of ginseng can have various components, which influence the curative effect. Thus, characteristic detection and quality control are needed for ginseng products in clinical applications.

## Figures and Tables

**Table 1:** Ginseng materials mentioned in this study.

Types	Samples	Processing	Main Components (mg/g Dry Weight)	Ref.
White ginseng	WKGE	Shade dried, Soxhlet extracted by water	1.93 Re, 1.91 Rc, 1.81 Rb2, 1.56 Rb1, 1.24 Rg1, 0.6 Rf, (×10^−3^)	[36]
	KWG	Air dried	9.1 Re, 3.0 Rg1, 2.4 Rb1, 1.3 Rc, 0.7 Rb2	[37]
	TCMGAR	Air dried	11.2 Rg3, 4.2 Rd, 4.1 Rb1, 3.8 Rh2, 2.3 Rb2, 2.0 Rc	[38]
	AG	Air dried	96.7 total ginsenosides (PPD:PPT = 3.03:1)	[26]
Red ginseng	KRG	Steamed and air dried	1.93 Rb1, 1.3 Rg3, 1.0 Rd, 0.93 Rb2	[39]
	KRG	Steamed at 90–100 °C; for 3 h and air dried	51.6 Rb1, 28.9 Rg1, 22.2 Rc, 21.6 Re, 18.2 Rb2	[22]
	KRG	Steamed and air dried	2.43 Rb1, 1.58 Rg1, 0.95 Rc, 0.89 Rb1, 0.62 Re	[40]
	KRG	Steamed and air dried	4.6 Rb1, 2.8 Rc, 2.3 Rb2, 1.4 Rg2, 1.2 Rg3, 1.2 Re	[41]
	KRG	Steamed and air dried	8.03 Rb1, 3.29 Rc, 2.80 Rb2, 2.50 Rg3, 1.47 Rf, 1.29 Re, 1.18 Rg1, 1.0 Rd	[24]
	KRG	Steamed and air dried	16.58 total ginsenosides (PPD:PPT = 1.65:1)	[20]
	ARG	Steamed in autoclave and dried	Related fatty acids, 58.1 cinnamic acid, 50.1 ferulic acid	[42]
Black ginseng	BGE	Steamed and dried, repeat several cycles, extracted by 70% ethanol at 70 °C for 12 h	5.6 C-K, 4.7 Rg5, 1.7 Rg3, 1.5 Rb1, 0.8 Rg2, 0.7 Rc	[30]
	GBG05-FF	Repeated steaming at 95 °C for 6 h and drying at 60 °C, extracted by 70% ethanol at 80 °C for 8 h	11.7 Rg5, 6.9 Rk1, 5.2 Rg3, 1.9 Rh4	[43]
Fermented ginseng	FRG	RGE incubated with yeast at 40 °C for 12 h	4.9 Rg3, 4.8 Rb1, 3.4 Rb2, 2.9 Rg2, 1.8 Re, 1.4 C-K, 0.8 Rg1	[44]
	FRG	RGE incubated with *L. plantarum* at 35–40 °C for 15 d	4.9 C-K, 3.5 Re, 3.3 Rb1, 3 Rb2, 2.4 Rd, 2 Rc	[21]
	FSGB	SGB incubated with *L. plantarum* at 30 °C for 72 h	Quinic acid, linoleic acid, palmitic acid	[45]
	FGE	GE incubated with microorganism	61.0 C-K, 27.7 Rg3, 12.1 Rh1, 9.5 Rd, 8.2 Rg2, 3.1 Rh2, 2.3 Rb2	[46]
	GS-E3D	RGE incubated with 10% pectin lyase at 50 °C for 5 d	30.2 Rb1, 17.6 Rb2, 14.0 Rc, 12.6 Re, 5.9 Rg1, 4.7 Rf, 2.7 Rg3, 1.5 Rg5	[47]
	VEG	GE incubated with vinegar (pH 2.3) at 90 °C for 6 h	40.5 Rg3, 4.1 Rc, 4.0 Rd, 1.9 Rb2, 1.1 Rf	[15]
	HGE	GE incubated with an enzyme solution	7.54 Rg1, 6.3 C-K, 5.42 Rb1, 1.87 Re, 0.70 Rd	[16]
Fractioned ginseng	Rg3-RGE	RG multiple extracted by 55% ethanol	51.7 Rg3, 3.86 Rb1, 3.71 Rh1, 3.55 Rg2, 1.6 Rd, 1.53 Rb2	[48]
	GB	Removed seeds and air dried, refluxed with 70% ethanol for 10 h	110.6 Re, 21.1 Rc, 19.0 Rb2, 16.6 Rg1, 16.5 Rd, 8.4 Rg2, 7.7 Rb1	[19]

**Table 2:** Effects of ginseng on diabetes-related parameters in human studies.

Material	Design (Sample Size and Subjects)	Drug Treatment and Duration	Results								Ref.
			BW	HbA1c	FBG	FI	PG	PI	HOMA-IR	Safety	
Vinegar extract of ginseng (VEG)	RCT (72 type 2 diabetic patients)	Four group (*n* = 18/group): 1500, 2000, 3000 mg of VEG, or placebo daily; 8 weeks	#	+	+	+	+	#	#	*	[15]
Korean red ginseng (KRG)	RCT (50 obese women)	6 g/d of KRG *n* = 24, or placebo *n* = 26; 8 weeks	+	#	-	#	#	#	#	#	[23]
Korean red ginseng (KRG)	Multicenter, RCT (1000 healthy adults)	2 g/d of KRG *n* = 495, or placebo *n* = 505; 24 weeks.	-	#	#	#	#	#	#	*	[24]
Korean red ginseng (KRG)	RCT (68 obese participants without diabetes)	6 g/d of KRG *n* = 34, or placebo *n* = 34; 12 weeks	-	#	-	-	#	#	-	#	[22]
Korean red ginseng (KRG)	RCT (41 type 2 diabetic patients)	5 g/d of KRG *n* = 21, placebo *n* = 20; 12 weeks	-	-	+	+	+	+	+	#	[20]
Fermented red ginseng (FRG)	RCT (42 impaired fasting glucose or type 2 diabetic patients)	2.7 g/d of FRG *n* = 21, placebo *n* = 21; 4 weeks	#	#	-	-	+	+	#	*	[21]
Hydrolyzed ginseng extract (HGE)	RCT (23 impaired fasting glucose participants)	960 mg/d of HGE *n* = 12, placebo *n* = 11; 8 weeks	-	#	+	-	+	-	-	*	[16]
Korean white ginseng (KWG)	RCT crossover trial (25 type 2 diabetic patients)	1 g, 3 g, 6 g KWG, or 3 g placebo together with 50 g glucose-load, acute test	#	#	-	#	-	#	#	*	[37]
Ginseng berry extract (GBE)	RCT (72 participants)	1 g/d GBE *n* = 34, placebo *n* = 38; 12 weeks	-	-	+	-	+	-	-	*	[19]
American ginseng (AG)	RCT parallel trial (74 type 2 diabetic patients)	3 g/d of AG *n* = 35, placebo *n* = 39; 12 weeks	#	#	#	#	#	#	#	*	[27]
American ginseng (AG)	RCT crossover trial (39 type 2 diabetic patients)	6 g/d of fiber from KGB together with 3 g AG; 12 weeks	-	+	-	-	#	#	#	*	[25]
American ginseng (AG)	RCT crossover trial (24 type 2 diabetic patients)	3 g/d AG or placebo with original treatment; 8 weeks	-	+	+	-	#	#	#	*	[26]

BW: body weight; FBG: fasting blood glucose; FI: fasting insulin; HbA1c: hemoglobin A1c; HOMA-IR: homeostasis model assessment-insulin resistance; PG: postprandial glucose; PI: postprandial insulin; RCT: randomized controlled trial. +: indicates the positive outcome with significant difference compared to the control (*p* < 0.05); -: indicates no significant differences compared to the control; #: indicates not tested in the literature; *: indicates the safety profiles were unaffected.

**Table 3:** Effects of ginseng on diabetes-related parameters in animal studies.

Material	Dose [Route of Administration]	Duration	Animal	Molecular Mechanism	Ref.
Ginseng extracts					
Fermented steam-dried ginseng berry (FSGB)	0.5 g/kg [ig]	7 wk	*db/db* mice	Decreased the blood glucose and body weight; increased the immune cell population and GLUT1 expression.	[45]
Ginseng berry (GB)	100, 200 mg/kg [ig]	10 wk	STZ-induced mice	Enhanced beta-cell proliferation and glucose tolerance, decreased blood glucose.	[28]
Tissue culture raised mountain ginseng adventitious root (TCMGARs)	125, 250, 500 mg/kg [diet]	4 wk	STZ-induced rats	Significantly reduced the blood glucose, TC, and TG levels.	[38]
Ginseng berry (GB)	0.05% [diet]	6 m	C57BL/6 mice (15 months old mice)	Increased the parameters of insulin sensitivity, IRS, AKT, and FOXO1; decreased PPAR-γ.	[29]
Wild Korean ginseng extract (WKGE)	100, 200, 300 mg/kg [ig]	8 wk	STZ-induced rats	Significantly reduced blood glucose, ALT and alkaline phosphatase levels.	[36]
Black ginseng extract (BGE)	50, 100, 200 mg/kg [ig]	5 wk	STZ-induced mice	Reduced hyperglycemia and NF-κB; increased the insulin/glucose ratio and β-cell function.	[30]
Black ginseng ethanol extract (GBG05-FF)	300, 900 mg/kg [diet]	4 wk	*db/db* mice	Reduced the parameters of fasting blood glucose, glucose tolerance, HbA1c, TG, TC levels, and lipid accumulation; enhanced the phosphorylation of the AMPK, and up-regulated the expressions of GLUT2 and GLUT4.	[43]
Red ginseng (RG)	100, 200 mg/kg [ig]	8 wk	ICR mice for type 1 (STZ induced) *db/db* mice for type 2	Improved the threshold shift of hearing, delayed latencies, and signal intensity decrease in type 2 diabetic mice; changes with no significance in type 1 diabetic mice.	[41]
Korean red ginseng (KRG)	200 mg/kg [ig]	10 wk	STZ-induced rats	39 genes were upregulated more than two-fold; 84 genes were down-regulated.	[49]
Pectin lyase-modified red ginseng extract (GS-E3D)	25, 50, 100 mg/kg [ig]	6 wk	STZ-induced rats	Decreased the urinary levels of albumin, 8-OHdG, and AGEs; suppressed oxidative stress.	[47]
Rg3-enriched red ginseng extract (Rg3-RGE)	2.5, 5 mg/kg [ig]	12 wk	LDL^-/-^ mice	Reduced the levels of glucose, TG, LDL, AST, and AST; inhibited atheroma formation.	[48]
Korean red ginseng (KRG)	200 mg/kg [diet]	12 wk	Goto Kakizaki rats	Reduced blood glucose, PTP-1B, UCP-2, and PARP; enhanced the production of GLUT-4 and insulin.	[40]
Fermented red ginseng (FRG)	0.5%, 1% [diet]	16 wk	*ob/ob* mice	Decreased body weight, blood glucose, and hyperlipidemia; increased the expressions of IR, LPL, GLUT1, GLUT4, PPAR-γ, and PEPCK.	[44]
Red ginseng extract (RGE)	2 g/kg [ig]	5 wk	STZ-induced rats	Metformin or RGE administered alone can reduce the FBG, but co-administration recovered the FBG to the control level.	[39]
Fermented ginseng extract (FGE)	0.1% *w*/*w* [diet]	8 wk	*db/db* mice	Decreased blood glucose, HbA1c, TNF-α, and lymphocytes; increased adiponectin, serum insulin, PPAR-γ2, and GLUT-2.	[46]
Ginseng polysaccharides	0, 12.5, 25, 50, 100, 200 mg/kg [ig]	10 d	STZ-induced ICR mice	Increased the concentration of insulin, SOD, and glycogen; decreased the content of MDA.	[50]
Ginseng powder	150 mg/kg [ig]	7 d	STZ-induced rats	Enhanced the expression of PPAR-δ and the phosphorylation of troponin1.	[51]
American red ginseng (ARG)	150 mg/kg [ip]	30 d	*db/db* mice	Reduced blood glucose, plasma cholesterol and LDL; increased glycogen and HDL.	[42]
American ginseng	200 mg/kg [ig]	2 m (type 1), 4 m (type2)	C57BL/6 mice for type 1 (STZ- induced), *db/db* mice for type 2	Increased stroke volume, ejection fraction, cardiac output, and left ventricle pressure; reduced oxidative stress.	[52]
American ginseng saponin (PQS) of stem and leaf	30, 60 mg/kg [ig]	8 wk	STZ-induced rats	Up-regulated the expressions of HGF, NO, ET-1, TNF-α, and slCAM-1.	[53]
Ginsenosides					
Panaxatriol	11 mg/d + 45 min/d (15 m/min) exercise [diet]	6 wk	KK-Ay/Ta Jcl (KKAy) mice	Significantly lowered blood glucose; no significant differences in body weight; significantly improved insulin resistance.	[54]
Rb1	10 mg/kg [ip]	1 wk	C57BL/6 mice (high fat)	Decreased fasting blood glucose, glucose tolerance, and 11β-HSD1; increased insulin sensitivity.	[31]
Re	5, 10, 20 mg/kg [ig]	4 wk	C57BL/6 mice (high fat)	Decreased the parameters of TG, TC, LDL-C, GOT, and GPT; increased HDLC, regulating ACh, AChE, MDA, SOD, and GSH via JNK pathway.	[55]
Rg1	10 mg/kg [ip]	4 wk	STZ-induced rats	Improved angiogenesis, increased eNOS activation, up-regulated VEGF expression, and inhibited apoptosis.	[56]
Rg1	10,15, 20 mg/kg [ip]	12 wk	STZ-induced rats	Decreased the parameters of cTnI, CK-MB, MDA, apoptosis, and CASP3; increased expressions of SOD, CAT, GSH, and Bcl-xL.	[57]
Rg1	10,15, 20 mg/kg [iv]	12 wk	STZ-induced rats	Reduced the expressions of cTnI, GRP78, and CHOP; inhibited endoplasmic reticulum stress-induced apoptosis.	[58]
Rg1	56.25 μM/kg [iv]	10 d	STZ-induced rats	Reduced the cerebral infarction volume; promoted neuronal recovery.	[59]
Rg1	25, 50 mg/kg [ig]	8 wk	STZ-induced rats	Reduced blood glucose levels and insulin resistance index; increased the parameters of TC, TG, LDL-C, AST, and ALT.	[32]
Rg3	0.5 mg/kg [ig]	Once	*db/db* mice	Increased the production of GLP-1 and insulin; decreased blood glucose.	[33]
Rh2	5 mg/kg [iv]	4 wk	STZ-induced rats	Decreased fasting blood glucose, ratio of heart weight/body weight, and PPAR-δ; increased STAT3, CCN2, and fibronectin.	[60]
Malonyl ginsenosides (MGR)	50, 100 mg/kg [ip]	3 wk	STZ-induced rats (high fat)	Significantly lowered FBG levels; reduced TG and TC; increased glucose disposal; no effect on body weight.	[61]
Ginsenoside Rg1 + Astragaloside IV	(50 Rg1 + 16 IV) mg/kg [ig]	8 wk	STZ-induced rats	Lowered MDA level and increased levels of CAT, GSH-PX, and T-AOC. Significantly reduced the levels of BUN, SCr, β_2_-MG, and UCr. Diminished the mRNA overexpression of TGF-β1 and CTGF; increased Smad7 expression.	[62]
Rg3	10 mg/kg/2 days [ip]	4 wk	STZ-induced ApoE^-/-^ mice	20(*S*)-Rg3 showed stronger anti-proliferative and anti-migratory effects than 20(*R*)-Rg3; (*S*)-isomer significantly reduced the plaque size of diabetic atherosclerosis.	[63]
Rg3	0.5 mg/kg [ig]	3 m	STZ-induced rats (high fat)	Inhibited inflammatory response (inhibited the expression of TGF-β_1_, IL-1, IL-6, IL-12) and promoted the activation of PI3K and MAPK signaling pathways to prevent lung tissue damage induced by hyperglycemia.	[64]

11β-HSD1: 11β-hydroxysteroid dehydrogenase type I; 8-OHdG: 8-hydroxy-2′-deoxyguanosine; Ach: acetylcholine; AChE: acetylcholinesterase; AGEs: advanced glycation end-products; AKT: protein kinase B; ALT: alanine aminotransferase; AMPK: 5’ adenosine monophosphate-activated protein kinase; AST: aspartate aminotransferase; Bcl-xL: B-cell lymphoma-extra-large; BUN: blood urea nitrogen; CASP3: caspase-3; CAT: catalase; CCN2: connective tissue growth factor; CHOP: C/EBP homologous protein; CK-MB: creatinine kinase MB; CTGF: connective tissue growth factor; cTnI: cardiac troponin I; eNOS: endothelial nitric oxide synthase; ET-1: endothelin-1; FBG: fasting blood glucose; FOXO1: forkhead box protein O1; GLP-1: glucagon-like peptide-1; GLUT1: glucose transporter 1; GLUT-2: glucose transporter protein 2; GLUT-4: glucose transporter protein 4; GOT: glutamic-oxaloacetic transaminase; GPT: glutamic-pyruvic transaminase; GRP78: glucose-regulated protein 78; GSH (-PX): glutathione peroxidase; HbA1c: hemoglobin A1c; HDL: high- density lipoprotein; HDL-C: high-density lipoprotein cholesterol; HGF: hepatocyte growth factor; IL: interleukin; IR: insulin receptor; IRS: insulin receptor substrate; JNK: c-Jun N-terminal protein kinase; LDL: low-density lipoprotein; LDL-C: low-density lipoprotein cholesterol; LPL: lipoprotein lipase; β2-MG: β2-microglobulin; MAPK: mitogen-activated protein kinase; MDA: malondialdehyde; NF-κB: nuclear factor–κB; NO: nitric oxide; PEPCK: phosphoenolpyruvate carboxykinase; PI3K: phosphoinositide 3 kinase; PPAR-γ2: peroxisome proliferator-activated receptor gamma 2; PPARδ: peroxisome proliferator-activated receptors δ; PTP-1B: protein tyrosine phosphatases-1B; SCr: serum creatinine; Sicam-1: soluble intercellular adhesion molecule 1; SOD: superoxide dismutase; STAT3: signal transducer and activator of transcription 3; STZ: streptozotocin; T-AOC: total anti-oxidative capacity; TC: total cholesterol; TG: triglycerides; TGF-β: transforming growth factor beta; TNF-α: tumor necrosis factor alpha; UCP-2: uncoupling protein 2; UCr: urinary creatinine; VEGF: vascular endothelial growth factor. ip: intraperitoneal injection; iv: intravenous injection; ig: intragastric injection.

**Table 4:** Effects of ginseng on diabetes-related molecular targets in cell line studies.

Material	Cell Line	Drug Dose	Molecular Mechanism	Ref.
Ginseng total saponin	Mouse podocytes	1 µg/mL for 6, 24, and 48 h	Ginseng total saponins improved p130Cas protein of podocytes.	[65]
Ginsenoside Rg3	Human NCI-H716	10 µM	Enhanced secreting of GLP-1.	[33]
Ginsenoside Rg3	Mouse islet cell	4 µM	Increased glucose-induced insulin secretion (2.3-fold higher); enhanced islet function and attenuated apoptosis.	[34]
Ginsenoside Rg3	Human pulmonary cell BEAS-2B	25, 50, 75, 100 μg/mL	No effect on the viability of BEAS-2B cells; high concentration could induce apoptosis compared with the control group.	[64]
Ginsenoside Rg1	C2C12 muscle cell	10, 20, 40 µM	Enhanced glucose uptake and GLUT4 by AMPK pathways.	[66]
Ginsenoside Re	3T3-L1 cell	3, 10, 30, 60 µM	Increased glucose uptake and the expressions of PPAR-γ2, IRS-1, ap2, and adiponectin genes expressions; helped the translocation of GLUT4 to the membranes; inhibited the expression and production of TNF-α.	[67]
Ginsenoside F2	3T3-L1 cell	10, 50, 100 µM	Reduced the content of lipid accumulation; down-regulated expression of PPAR-γ and perilipin.	[68]
Ginsenoside Rg5:Rk1 mixture	3T3-L1 cell	10, 100 µg/mL	Inhibited lipid accumulation and reduced TG level. Decreased the mRNA level of STAT3, PPAR-γ, CEBPα, and ap2; reduced protein expression of PPAR-γ and CEBPα.	[69]

AMPK: adenosine 5’-monophosphate (AMP)-activated protein kinase; ap2: activating protein; CEBPα: CCAAT/enhancer binding protein alpha; GLP-1: glucagon-like peptide-1; GLUT4: glucose transporter protein 4; IRS-1: insulin receptor substrate 1; PPAR: peroxisome proliferators-activated receptors; STAT3: signal transducer and activator of transcription 3; TNF: tumor necrosis factor.
